# Immunodiagnosis of Canine Visceral Leishmaniasis Using Mimotope Peptides Selected from Phage Displayed Combinatorial Libraries

**DOI:** 10.1155/2015/401509

**Published:** 2015-01-29

**Authors:** Christina Monerat Toledo-Machado, Ricardo Andrez Machado de Avila, Christophe NGuyen, Claude Granier, Lilian Lacerda Bueno, Claudia Martins Carneiro, Daniel Menezes-Souza, Rubens Antonio Carneiro, Carlos Chávez-Olórtegui, Ricardo Toshio Fujiwara

**Affiliations:** ^1^Departamento de Parasitologia, ICB, Universidade Federal de Minas Gerais, CP 486, Belo Horizonte 31270-901, MG, Brazil; ^2^Departamento Bioquímica e Imunologia, ICB, Universidade Federal de Minas Gerais, CP 486, Belo Horizonte 31270-901, MG, Brazil; ^3^SysDiag CNRS-BioRad UMR 3145, Cap Delta/Parc Euromédecine, 1682 rue de la Valsière, CS 61003, 34184 Montpellier Cedex 4, France; ^4^Departamento de Análises Clínicas, Escola de Farmácia, Universidade Federal de Ouro Preto, Ouro Preto 35400-000, MG, Brazil; ^5^Escola de Veterinária, Universidade Federal de Minas Gerais, CP 486, Belo Horizonte 31270-901, MG, Brazil

## Abstract

ELISA and RIFI are currently used for serodiagnosis of canine visceral leishmaniasis (CVL). The accuracy of these tests is controversial in endemic areas where canine infections by *Trypanosoma cruzi* may occur. We evaluated the usefulness of synthetic peptides that were selected through phage display technique in the serodiagnosis of CVL. Peptides were chosen based on their ability to bind to IgGs purified from infected dogs pooled sera. We selected three phage clones that reacted only with those IgGs. Peptides were synthesized, polymerized with glutaraldehyde, and used as antigens in ELISA assays. Each individual peptide or a mix of them was reactive with infected dogs serum. The assay was highly sensitive and specific when compared to soluble *Leishmania* antigen that showed cross-reactivity with anti-*T. cruzi* IgGs. Our results demonstrate that phage display technique is useful for selection of peptides that may represent valuable synthetic antigens for an improved serodiagnosis of CVL.

## 1. Introduction

Canine visceral leishmaniasis (CVL) caused by* Leishmania (Leishmania) infantum chagasi *is a widespread zoonotic disease of both the Old and the New World [1] leading to a considerable number of deaths. Domestic dogs are considered the main animal reservoir hosts of the disease [2, 3]. Most infected dogs do not present clinical signs but are seropositive particularly in endemic areas of CVL in the World [2, 4–7]. The seroprevalence of CVL in areas of endemicity in the Mediterranean Basin and the Middle East, including Iran, has been reported to be 10–37% [6, 8]. Euthanasia of seropositive dogs has been adopted as a mainstay control measure in some countries [9].

The Brazilian Ministry of Health recommends the use of an immunoenzymatic assay (ELISA) and an indirect immunofluorescence antibody test (IFAT) for the diagnosis of canine visceral leishmaniasis (CVL), employed as criteria for the culling of seropositive dogs in surveillance and control programs for visceral leishmaniasis (VL) [10]. Both the accuracy of these tests and the process of dog culling promote a controversial impact of the leishmaniasis infection [11, 12]. However, the development of an effective diagnosis test can be critical for the control and the possible eradication of VL; more sensitive and specific tests may be especially helpful to achieve this goal [13].

Over the past years, synthetic peptides have been used successfully as antigens for the* in vitro* diagnosis of many parasitic diseases [14]. Phage display of random peptides has become an alternative method for the study of molecular interactions in many areas of protein science, including antigen-antibody interactions. It has been shown that linear epitopes, as well as mimotopes that mimic discontinuous epitopes of an antigen, can be identified by the screening of phage libraries with monoclonal or polyclonal antibodies [15, 16]. Phage display in neglected disease research has proven successful not only in mapping the protein-protein interactions that are important in the etiologic agent biology, but also in the identification of molecules that might be exploited in the design of therapeutic agents, vaccines, or immunodiagnostics [17–19].

In order to search for diagnostic epitopes without previous knowledge of protein structure, we tested phage-borne libraries displaying foreign peptides at the surface of the major pVIII coat proteins for their capacity to bind anti-*L. infantum chagasi *proteinantigen (LiPA) IgGs purified from the sera of dogs with visceral leishmaniasis. Phage clones reactive with anti-LiPA IgGs were also tested for reactivity with IgGs purified from the sera of dogs experimentally infected with* T. cruzi*, because of the known cross reaction between visceral leishmaniasis antibodies and Chagas' disease antibodies in dogs [20]. In order to estimate the diagnostic accuracy, we found three peptides that could successfully be used as antigens in ELISA assays for a specific immunodiagnosis of canine visceral leishmaniasis without cross reaction with circulating antibodies of* T. cruzi* experimentally infected dogs.

## 2. Material and Methods

### 2.1. Study Dogs

For biopanning assay thirty-eight sera from* L. infantum chagasi *naturally infected dogs of both genders were used. For the ELISA assays, thirty-eight sera were obtained from beagle dogs experimentally infected with* L. infantum chagasi *for leishmaniasis studies. The infection was certified through parasitological tests that were conducted on bone marrow cells examined by optical microscopy and immunological tests (ELISA and RIFI). Additionally, for both assays, thirty-eight sera from uninfected dogs were used as negative controls. In order to evaluate cross-reactivity on ELISA assays we also used fifteen sera from dogs experimentally infected with* T. cruzi* parasite obtained from the serum bank of the Laboratório de Imunologia e Genômica de Parasitos (UFMG).

Proof of dog infection was attested by a positive immunofluorescence titre (IFAT) at the threshold titer of 1 : 40 serum dilution, a positive reactivity in ELISA, and a parasitological diagnosis of* Leishmania*. Antibodies from sera of 38 healthy dogs displaying negative IFAT and parasitological tests were included as uninfected controls. Briefly, the clinical evaluation assessed typical clinical signs for symptomatic visceral leishmaniasis, including lymphadenopathy, decrease of weight and opaque eye, alopecia, eczema, and skin ulcers.

In order to verify possible infection with different pathogens (*Babesia canis*,* Ehrlichia canis, and Trypanosoma cruzi*) in all* L. infantum chagasi* naturally infected animals, parasitological exams were performed in a private laboratory in Belo Horizonte, Minas Gerais, Brazil.

All dogs were maintained in a kennel of Institute of Biological Sciences in the Universidade Federal de Minas Gerais, Belo Horizonte, Brazil, according to university's ethic committee for clinical research (CETEA) protocol 122/2009.

### 2.2. Production of* L. infantum chagasi* Protein Antigen (LiPA)

*L. infantum chagasi* (MHOM/BR/1975/BH46) was grown at 24°C in Schneider's medium (Sigma, St. Louis, MO, USA) supplemented with 20% heat-inactivated fetal bovine serum (FBS; Sigma), 200 U/mL penicillin, and 100 *μ*g/mL streptomycin, pH 7.2. Protein antigens of* L. infantum chagasi* (LiPA) were prepared from stationary phase promastigotes, submitted to 7 cycles of freezing (liquid nitrogen) and thawing (42°C), followed by ultrasonication (Ultrasonic processor, GEX600) with cycles of 10 sec for 2 min at 35 MHz. The extracts were then submitted to centrifugation at 8,000 ×g for 20 min at 4°C. The supernatant was collected and stored at −70°C. The protein concentration was estimated by the Bradford method [21].

### 2.3. Preparation of Antibodies for Biopanning

Antibodies used for biopanning (i.e., immunocapture of phages binding to target antibodies) were initially purified from 38 sera from* L. infantum chagasi *naturally infected dogs of both genders. Fifteen serum samples of dogs experimentally infected with* Trypanosoma cruzi* parasite were also collected.

Polyclonal IgGs to LiPA (anti-LiPA IgGs) used for biopanning were purified from a pool of dogs with VL, using ammonium sulfate precipitation and filtration through Protein A-Sepharose 4B column [22]. Following elution and neutralization using NaOH 0.1 M, the IgG fraction was dialyzed against PBS 1x and the protein concentration was determined by the Bradford method [21]. IgGs from uninfected controls (normal IgG) were also fractionated as described before. IgGs from* T. cruzi* infected dogs were obtained. The reactivity against LiPA of anti-LiPA IgGs and normal IgGs was confirmed by indirect ELISA.

### 2.4. Ethics Statement

All sera samples were obtained from the Veterinary Hospital of the Federal University of Minas Gerais (UFMG) and the experiments were performed in compliance with the university's ethic committee for clinical research (CETEA), protocol 122/2009. All sera were stored at −20°C until use. All dog owners gave permission to have their animals sampled.

### 2.5. Biopanning

The M13 phage libraries expressing 15-mer (*X*_15_) and 12-mer peptides including two fixed cysteine residues (*X*C*X*_8_C*X*) were previously described by [23] and obtained from Dr. John Scott (Simon Fraser University, Burnaby, British Columbia, Canada). Three cycles of biopanning were performed as described by Ferrières et al. (2000) [24]. Anti-LiPA IgGs were coated onto a polystyrene Petri dish (10 × 1.5 cm, Falcon 1029) overnight at 4°C at a concentration of 5 *μ*g/mL for the first two rounds of panning and a concentration of 0.5 *μ*g/mL for the last panning in 100 mM NaHCO_3_, pH 8.6, on a shaking platform. For the first panning, 5 × 10^12^ transducing units (TU) of each library were incubated with the absorbed IgG. After incubation, unbound phages were washed with TBS (Tris 50 mM, NaCl 150 mM, pH 7.5) containing 0.05% (v/v) Tween 20, and bound phages were removed by elution with 0.1 M glycine, pH 2.2, containing 0.1% BSA. Eluted phages were then used to infect* Escherichia coli *K91 cells. After three rounds of enrichment, individual phage clones were isolated and further analyzed [24].

### 2.6. ELISA Analysis after Three Rounds of Panning

ELISA plates (Falcon 3912, Becton Dickinson, Oxnard, CA 93030) were coated with 1 *μ*g/well of anti-LiPA IgGs in 100 mM NaHCO_3_, pH 8.6, and overnight at 4°C. Plates were washed with PBS 0.1% Tween 20 (v/v) and then blocked with PBS, 0.1% Tween 20 and 2% nonfat dried milk (w/v) for 1 h at 37°C. 10^10^ TU of phages, eluted after each round of panning, were then added to the plate and incubated for 2 h at 37°C. Binding was detected using a peroxidase conjugated anti-M13 antibody (Roche Molecular Biochemicals) diluted 1 : 3000 in blocking buffer. After 1 h at 37°C and washing, the peroxidase substrate was added. The resulting color was measured at 492 nm with an automated microtiter plate reader (Model 450, Bio-Rad).

### 2.7. Screening

ELISA plates (Falcon 3912, Becton Dickinson, Oxnard, CA 93030) were coated with 1 *μ*g/well of anti-LiPA IgGs or anti-* T. cruzi* IgGs in 100 mM NaHCO_3_, pH 8.6, and overnight at 4°C. Plates were washed with PBS, 0.1% Tween 20 (v/v), and then blocked with PBS, 0.1% Tween 20, and 2% nonfat dried milk (w/v) for 1 h at 37°C. 10^10^ TU of individual phages was isolated after third panning and 50 *μ*L of blocking buffer was then added to each well. Phage particles were incubated for 2 h at 37°C. Binding was detected using a peroxidase conjugated anti-M13 antibody (Roche Molecular Biochemicals) diluted 1 : 3000 in blocking buffer. After 1 h at 37°C and washing, the peroxidase substrate was added. The resulting color was measured at 492 nm with an automated microtiter plate reader (Model 450, Bio-Rad). Afterwards, the twelve clones were checked by ELISA for their ability to bind to anti* T. cruzi *dog IgGs.

### 2.8. DNA Sequencing, Synthesis, Chromatography, and Mass Spectrometry of Soluble Peptides

Approximately, 9 *μ*g of single-stranded DNA was purified using the QIA prep Spin M13 protocol (Qiagen). Sequencing reactions were carried out according to the dideoxy chain termination method [25], using the ABI Prism Kit (PE Applied Biosystems) for the automatic method with ABI PRISM 377 (PerkinElmer). The primer reverse 5′-TCGGCAAGCTCTTTTAGG-3′ was used for sequencing. The sequences obtained were translated.

The peptides TTDDDKLKKTLTYRS, KCPSIPGAVLCV, and ICARQDPAGNCS were synthesized in ResPep SL Synthesizer by Fmoc chemistry [26]. After synthesis, the peptides were deprotected and released from the resin by trifluoroacetic acid (TFA) treatment in the presence of the appropriate scavengers. The peptides were lyophilized and their purity was assessed by HPLC and their mass was confirmed by mass spectrometry according to de Avila et al. (2011) [27].

### 2.9. Glutaraldehyde Link Reaction

Individual peptides and the combination of three peptides (pool) were polymerized using glutaraldehyde as cross-linking reagent [28]. Briefly, 10 *μ*moles of each peptide was diluted in 1 mL of PBS and an equal volume of 1% GLUT (20 mM) in PBS was then slowly added to the solution over the course of 1 hour under constant stirring at 4°C. The reaction was allowed to proceed for an additional hour and the free aldehyde coupling groups and the Schiff's base intermediates were reduced by the addition of NaBH_4_ (10 mg/mL). At the end of the reaction, the solution containing polymerized peptides was dialyzed overnight against PBS.

### 2.10. Diagnostic ELISA for Canine Sera

ELISA was carried out to detect circulating antibodies that recognized polymerized peptide in canine sera. ELISA assays microtiter plates were coated with 50 *μ*L/well of each polymerized peptide or with the polymerized mixture of the three peptides or with* L. infantum chagasi *total protein antigen (2.5 mg/mL) in Milli-Q water. The wells were blocked with 200 *μ*L/well of 5% BSA in PBS at 37°C for 1 h. After the wells had been washed twice with PBS containing 0.05% Tween 20 (PBS-T), 100 *μ*L/well of* L. infantum chagasi* infected dog serum; healthy dog serum; and* Ehrlichia canis*,* Babesia canis*, and* T. cruzi* infected dog serum diluted 1 : 100 in PBS- BSA 2.5% was added and incubated at 37°C for 1 h. The wells were washed six times with PBS-T, incubated with 100 *μ*L/well of anti-dog IgG conjugated with peroxidase (Sigma), diluted 1 : 2000 in PBS-BSA 2.5% at 37°C for 1 h and washed six times with PBST. After incubation with 100 *μ*L/well of o-phenylenediamine solution (0.33 mg/mL in citrate buffer, pH 5.2, in the presence of 0.04% hydrogen peroxide) for 15 min at room temperature, the optical density at 492 nm of each well was determined. The cut-off was determined according to ROC curve. The results of the ELISA peptides as antigens in dogs were compared with the immunoassay EIE-LVC kit (FIOCRUZ-Bio-Manguinhos, Brazil), which is the test currently recommended by the Brazilian Ministry of Health for screening seroreactive animals [29]. The assays were conducted according to the manufacturer's instructions.

### 2.11. Statistical Analysis

The lower limit of positivity (cut-off) was established for optimal sensitivity and specificity using the ROC (receiver operator curve) curve for all peptides used in this study and* L. infantum chagasi* antigen. The criterion to determine the cut-off value using the ROC curve was to select the point that showed higher sensitivity and specificity simultaneously. EIE-LVC^#^ cut-off was obtained according to the manufacturer (twice the average of the negative control provided by the kit). Sensitivity (Se), specificity (Sp), predictive positive value (PPV), and predictive negative value (PNV) were determined. The performance of each test was evaluated according to the area under curve (AUC) referent to the ROC curve and accuracy (AC). The degree of agreement between ELISA assays using peptides,* L. infantum chagasi* antigen, and EIE-LVC Bio-Manguinhos kit test was determined by the Kappa index (*κ*) values with 95% confidence intervals and interpreted according to the following Fleiss scale: 0.00–0.20, poor; 0.21–0.40, fair; 0.41–0.60, moderate; 0.61–0.80, good; 0.81–0.99, very good; and 1.00, perfect [30]. All of the statistical analyses were performed using GraphPad Prism (version 5.0) and GraphPad QuickCals (http://www.graphpad.com/quickcalcs/).

## 3. Results

### 3.1. Selection of Phages Recognized by Anti-LiPA IgGs and Mimotope Peptides Synthesis

**Figure 1:**
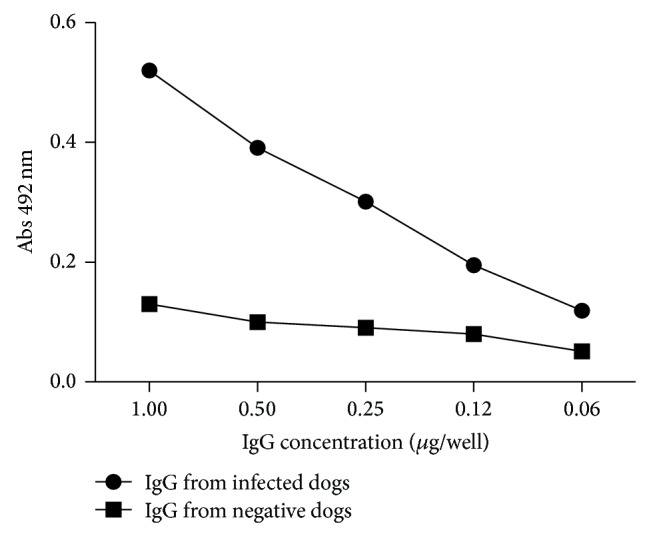
Reactivity of purified IgG antibodies against* L. infantum chagasi* antigen (LiPA). 10 *μ*g of affinity-purified IgGs from CVL dogs (●) and negative dogs (■) was added to each well (from 10 *μ*g to 0.625 *μ*g/well) of microtiter plates coated with 50 *μ*g/mL LiPA. The reaction was detected using a peroxidase conjugated anti-dog IgG antibody (1 : 2000). Absorbance values at 492 nm were means of duplicates.

For the screening of phage-borne peptides from libraries, IgGs purified from sera of thirty-eight naturally infected and sixteen uninfected dogs were used. Levels of anti-LiPA antibodies were previously examined in sera samples collected one week before phage display experiments (data not shown). After fractionation of the pool of sera of infected or noninfected animal through Protein A-Sepharose 4B column, a dose-dependent reactivity of the purified IgGs towards LiPA was observed in an ELISA format (Figure 1).

**Figure 2:**
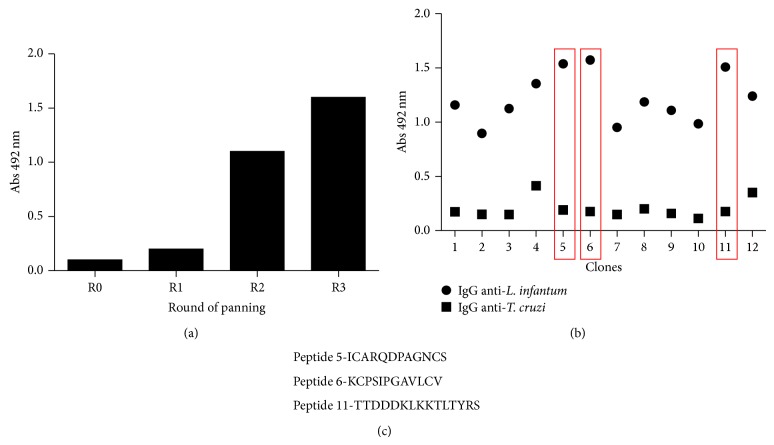
(a) Enrichment of phage binding after three rounds of panning. 50 *μ*L of a 10^10^ transduction unit phage suspension collected after each round of panning was added to wells of microtiter plates coated with 5 *μ*g/mL anti-LiPA IgG. Phages were detected using a peroxidase conjugated anti-M13 antibody (1 : 3000). Values of absorbance at 492 nm are means of duplicates. (b) Reactivity of individual clones isolated after panning 3. 50 *μ*L of a 10^10^transduction unit phage suspension isolated after the third round of panning was added to each well of microtiter plates coated with 1 *μ*g/well of anti-*L. infantum chagasi* IgGs (●) and anti-*T. cruzi* IgGs (■). Phages were detected using a peroxidase conjugated anti-M13 antibody (1 : 3000). Values of absorbance at 492 nm are means of duplicates. (c) The aminoacid sequences of the three selected peptides.

In order to identify peptides that bind to anti-LiPA antibodies, four different phage libraries were screened. A significant enrichment of phage binding to the target antibodies was obtained after three rounds of panning (Figure 2(a)). One hundred and ninety-eight phage clones were randomly picked from the third round of selection, and twelve clones were selected based on their reactivity (absorbance at 492 nm ≥ 1.0) against IgGs from* L. infantum chagasi* infected dogs (data not shown). Afterwards, the twelve clones were checked by ELISA for their ability to bind to anti-*T. cruzi *dog IgGs. Clones 5, 6, and 11 were further selected due their high reactivity against* Leishmania-*specific IgGs and without cross-reactivity with IgGs from dogs with Chagas disease (Figure 2(b)).

The DNA sequences of clones 5, 6, and 11 were translated and the amino acid sequences of the peptides were deduced (Figure 2(c)). One peptide (peptide 11) was selected from the *X*_15_ library expressing 15-mer linear peptides, whereas the other two peptides were selected from the* X*C*X*_8_C*X* library of constrained 12-mer peptides. The corresponding synthetic peptides, TTDDDKLKKTLTYRS, KCPSIPGAVLCV, and ICARQDPAGNCS, were chemically synthesized and purified by reverse phase chromatography and their correct molecular weights were confirmed by mass spectrometry (data not shown).

### 3.2. Sensitivity and Specificity of ELISA Tests with Synthetic Peptides for Serodiagnosis of CVL

**Figure 3:**
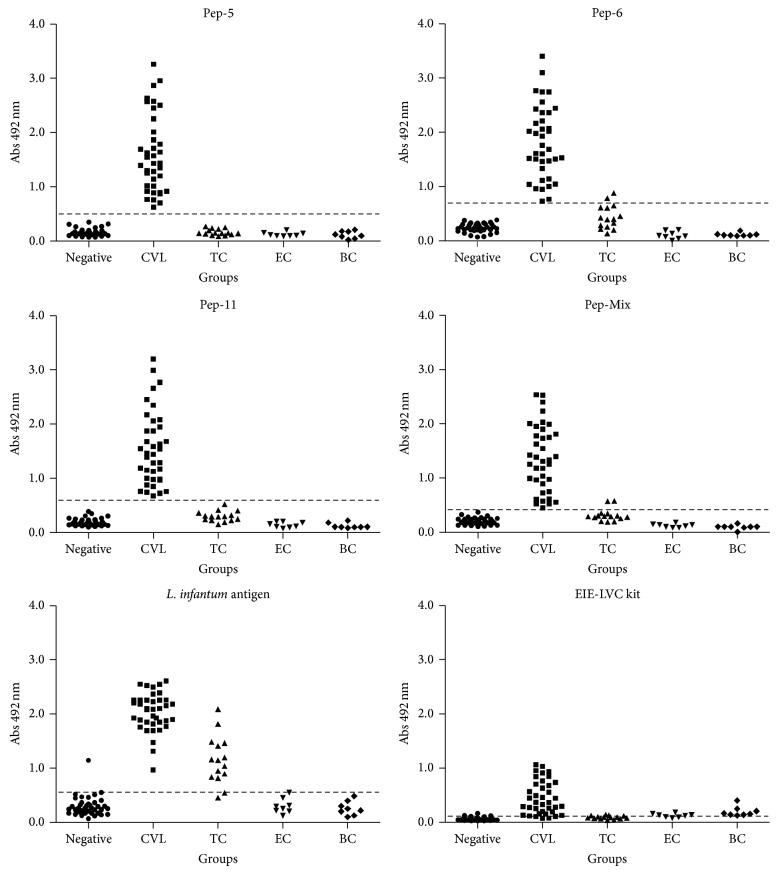
Comparison of ELISA reactivity of canine sera against individual phage-display selected peptides, the pool of peptides, the* L. infantum* chagasi antigen (LiPA), and reactivity in the EIE-LVC kit. ELISA was performed in different groups of dogs (negative/*control group*, CVL group, and TC/*T. cruzi* group). Cut-off was determined according to ROC curves.

In order to evaluate the antigen-specific dog antibody response against synthetic peptides, an ELISA was optimized to obtain the best signal-to-noise ratio and to develop a reproducible and robust assay capable of capturing antibodies over a biologically relevant assay range. The EIE-LVC kit was used as a reference. ELISA cut-off values for peptides 5, 6, and 11 and pooled peptides (peptides 5+6+11) as antigens were 0.488, 0.698, 0.594, and 0.410, respectively. In similar conditions, the cut-off values were 0.546 and 0.104, for* L. infantum chagasi* antigen and EIE-LVC kit, respectively. The peptides were initially tested on sera of 38 dogs with* L. infantum chagasi* (CVL) (Figure 3, Table 1). Antibodies against peptides 5, 6, and 11 and pooled peptides were detected in 100% of the serum samples from these dogs. In order to evaluate specificity (Sp), serum samples from negative control dogs and samples from dogs infected by* E. canis*,* B. canis*, and* T. cruzi* were tested. Peptides 5 and 11 demonstrated an excellent specificity value (100.00%) slightly better than the 97.10% obtained for peptide 6 and pooled peptides, which showed 2 false positive results in the* T. cruzi* group (Figure 3 and Table 1). Comparative analysis using either the crude antigen or the reference EIE-LVC ELISA demonstrated also high sensitivity (100.00% and 94.74%, resp.). However, these tests showed a poorer specificity (76.81% and 68.12%, resp.) due to the large number of false negative results in negative and* T. cruzi* group.

### 3.3. Measures of ELISA Performance and Analysis of Agreement between the Different Antigens Used in the Diagnosis of CVL

Measures of performance for the different tests evaluated are shown in Table 1. Maximum positive predictive value (PPV) was achieved by peptides 5 and 11 (100.00%) followed by peptide 6 and pool (95.00% for both). Excellent negative predictive values (NPV) were observed for all three peptides and their mix (100.00%).

**Figure 4:**
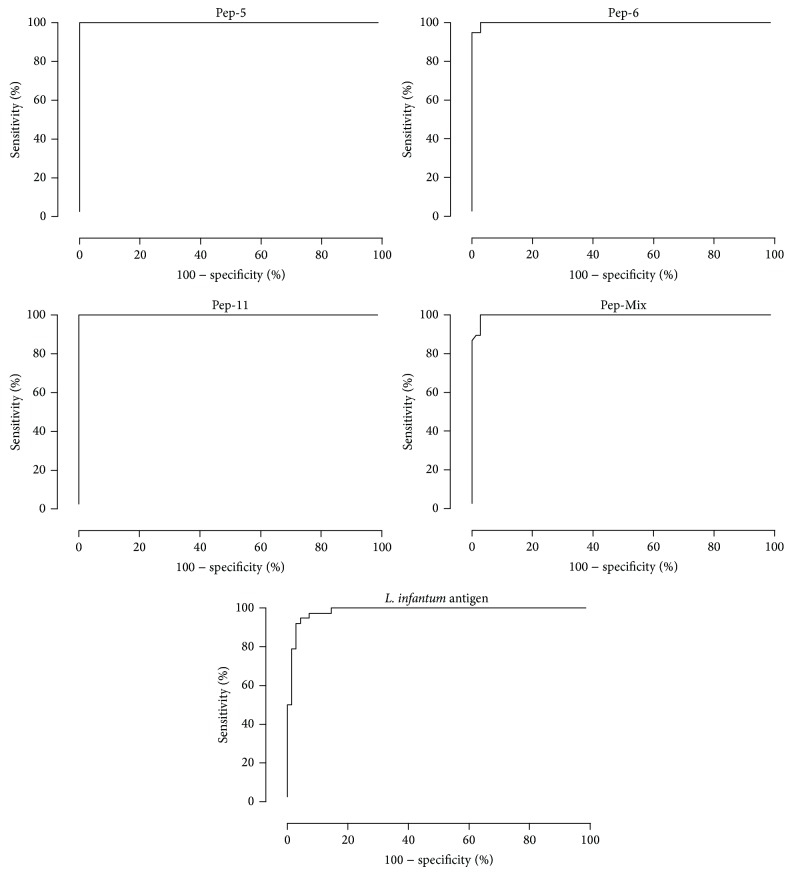
ROC curves obtained from all tests. The curves were used to determine ELISA cut-off, sensitivity, specificity, and AUC.

The area under the curve (AUC) and accuracy (AC) were used to compare the efficiency of different diagnostic tests [31]. Peptides 5 and 11 presented the highest AUC value (1.0000) and accuracy (AC = 1.0000), followed by peptide 6 (AUC = 0.9980 and AC = 0.9780) and pool (AUC = 0.9958 and AC = 0.9780) (Figure 4 and Table 1). The* L. infantum chagasi* antigen and EIE-LVC kit showed a lower accuracy value (0.8352 and 0.8791, resp.). The agreement between the serological tests using the Kappa Index is shown in Table 2. All peptides showed moderate agreement with EIE-LVC^∗^ and good agreement with crude leishmanial antigen.

## 4. Discussion

Accurate diagnosis of canine leishmaniasis is essential towards a more efficient control of this zoonosis in endemic areas where it occurs. Conventional parasitological techniques are highly specific but still represent time-consuming and invasive methods that are not appropriate for epidemiological surveillance or the daily routine of protocols for control of CVL. On the other hand, the use of rapid and reliable methods such serological assays for diagnosis of visceral leishmaniasis in dogs has been largely hampered by cross-reactivity with other canine pathogens [20, 32], especially when crude antigens are employed [33].

The use of synthetic peptides [34] as final targets in serological assays would circumvent the reliance on parasite extracts, which often do not provide reproducible results due to the variable nature of the protein content, thus contributing to the required standardization of the assay. Moreover, the chemical synthesis of the peptides is relatively simple and does not require the manipulation of pathogenic organisms. In the current study, we assessed the potential of phage display technology [15] to select specific peptide sequences and we further evaluated the antigenicity of selected epitopes for development of diagnostics of visceral leishmaniasis. In order to identify putative diagnostic epitopes, random peptide libraries displayed on the surface of M13 bacteriophages were used as repositories of molecular structures, some of which being supposed to mimic structural regions of* Leishmania *spp. antigens. The biopanning process led to identification of three peptides sequence (TTDDDKLKKTLTYRS, pep 11; KCPSIPGAVLCV, pep 6; and ICARQDPAGNCS, pep 5) from the* X15* and* X*C*X*_8_C*X* libraries. The comparison of the sequences of the selected peptides with two GenBank peptide sequence databases did not reveal any significant similarity with amino acid sequences of antigens previously characterized from* L. infantum chagasi*, suggesting that selected peptides might correspond either to yet unknown proteins or to conformational epitopes derived from tertiary/quaternary structures of* L. infantum chagasi* proteins. Of note, the two peptides with a disulfide bridge shared a similar motif (PGA for peptide 6 and PAG for peptide 5), which might correspond to a single epitope of an unknown* Leishmania* protein or a mimotope [29].

The cross-reactivity often described to crude parasite extracts [32, 33, 35–37] was also observed in our study showing that ELISA using* L. infantum chagasi* (LiPA) and the EIE-LVC ELISA kit recognized antibodies present in a large number of sera from dog affected by Chagas disease. In contrast, no positive response was observed when the same sera samples were tested using peptide-based (peptides 5 and 11) ELISA. Noteworthy, the ELISA carried out using synthetic peptides 5 and 11 presented a specificity of 100%, while peptide 6 or the pool of the three peptides rendered a specificity of considerable magnitude (97.10%). Nonetheless, the three peptides were able to detect all* Leishmania* positive samples (100% of sensitivity). These results using peptides selected by phage display for LVC immunodiagnosis tests showed better performance than other studies using crude parasite extracts [20, 32, 33, 38], recombinant antigens [35–37, 39], and even synthetic peptides [34]. Of note, in order to provide a better binding of the selected peptides to ELISA plates and enhance their antigenic properties, the mimotopes were covalently polymerized using glutaraldehyde (GLUT) as linker [40].

In summary, our data suggest that the three mimotope peptides identified by phage display would represent highly potential antigens to identify canine visceral leishmaniasis. Such antigens could be used alone or in combination with promising recombinant antigens in order to maximize the performance of the serological assays. Further studies using large cohorts of negative and positive individuals from endemic areas are still required to determine the use of these antigens (alone or in combination with different recombinant proteins) for control of CVL in endemic areas. Finally, our study suggests that mimotope-based ELISA strategy may be useful for the development of a sensitive and highly specific serodiagnosis for CVL or other parasitic diseases.

## Figures and Tables

**Table 1:** Diagnostic performance of synthetic peptides, *L*. *infantum chagasi* antigen, and EIE-LVC Kit in sera of dogs.

Diagnostic test	FN	FP	Se %	Sp %	PPV %	NPV %	AUC	AC
5^*^	0/38	0/69	100.00	100.00	100.00	100.00	1.0000	1.0000
6^*^	0/38	2/69	100.00	97.10	95.00	100.00	0.9985	0.9813
11^*^	0/38	0/69	100.00	100.00	100.00	100.00	1.0000	1.0000
Pep-Mix^*^	0/38	2/69	100.00	97.10	95.00	100.00	0.9968	0.9813
Ag^*^	0/38	16/69	100.00	76.81	70.37	100.00	0.9851	0.8505
EIE-LVC^#^	2/38	22/69	94.74	68.12	62.07	95.92	NA	0.7757

Samples from healthy dogs and dogs with canine visceral leishmaniasis, *T*. *cruzi*, *E*. *canis,* or *B*. *canis*.

^*^Cut-off obtained by ROC curve.

^#^Cut-off obtained according to the manufacturer.

NA: not applicable; FN: false negative; FP: false positive; Se: sensitivity; Sp: specificity; AUC: area under curve; PPV: positive predictive value; NPV: negative predictive value; AC: accuracy.

**Table 2:** Kappa index (*κ*) between paired results of diagnostic tests using peptides, *L. infantum chagasi* antigen, and EIE-LVC kit.

Diagnostic test	EIE-LVC^#^			Ag^*^		
	P	N	T	P	N	T
Pep-5^*^						
P	36	2	38	36	2	38
N	21	48	69	9	60	69
T	**57**	**50**	**107**	**45**	**62**	**107**
*κ* index (95% CI)	0.578 (0.435–0.721), moderate			0.784 (0.665–0.904), good		

Pep-6^*^						
P	37	3	40	40	0	40
N	20	47	67	14	53	67
T	**57**	**50**	**107**	**54**	**53**	**107**
*κ* index (95% CI)	0.557 (0.407–0.708), moderate			0.739 (0.616–0.862), good		

Pep-11^*^						
P	36	2	38	36	2	38
N	21	48	69	9	60	69
T	**57**	**50**	**107**	**45**	**62**	**107**
*κ* index (95% CI)	0.578 (0.435–0.721), moderate			0.784 (0.665–0.904), good		

Pep-Mix^*^						
P	37	3	40	40	0	40
N	20	47	67	14	53	67
T	**57**	**50**	**107**	**54**	**53**	**107**
*κ* index (95% CI)	0.557 (0.407–0.708), moderate			0.739 (0.616–0.862), good		

Ag^*^						
P	41	12	53			
N	16	38	54			
T	**57**	**50**	**107**			
*κ* index (95% CI)	0.477 (0.311–0.643), moderate					

Samples from healthy dogs and dogs with canine visceral leishmaniasis, *T*. *cruzi*, *E*. *canis,* or *B*. *canis*.

^*^Cut-off obtained by ROC curve.

^#^Cut-off obtained according to the manufacturer.

P: positive; N: negative; T: total; CI: confidence interval.
